# Genome-Wide Scan of Gastrointestinal Nematode Resistance in Closed Angus Population Selected for Minimized Influence of MHC

**DOI:** 10.1371/journal.pone.0119380

**Published:** 2015-03-24

**Authors:** Eui-Soo Kim, Tad S. Sonstegard, Marcos V. G. B. da Silva, Louis C. Gasbarre, Curtis P. Van Tassell

**Affiliations:** 1 Animal Genomics and Improvement Laboratory, United States Department of Agriculture, Agricultural Research Service, Beltsville, Maryland, United States of America; 2 Embrapa Gado de Leite, Empresa Brasileira de Pesquisa Agropecuária, Juiz de Fora, Brazil; Wageningen UR Livestock Research, NETHERLANDS

## Abstract

Genetic markers associated with parasite indicator traits are ideal targets for study of marker assisted selection aimed at controlling infections that reduce herd use of anthelminthics. For this study, we collected gastrointestinal (GI) nematode fecal egg count (FEC) data from post-weaning animals of an Angus resource population challenged to a 26 week natural exposure on pasture. In all, data from 487 animals was collected over a 16 year period between 1992 and 2007, most of which were selected for a specific *DRB1* allele to reduce the influence of potential allelic variant effects of the MHC locus. A genome-wide association study (GWAS) based on BovineSNP50 genotypes revealed six genomic regions located on bovine Chromosomes 3, 5, 8, 15 and 27; which were significantly associated (-*log_10_ p*=4.3) with Box-Cox transformed mean FEC (BC-MFEC). DAVID analysis of the genes within the significant genomic regions suggested a correlation between our results and annotation for genes involved in inflammatory response to infection. Furthermore, ROH and selection signature analyses provided strong evidence that the genomic regions associated BC-MFEC have not been affected by local autozygosity or recent experimental selection. These findings provide useful information for parasite resistance prediction for young grazing cattle and suggest new candidate gene targets for development of disease-modifying therapies or future studies of host response to GI parasite infection.

## Introduction

Nematode parasites are a primary animal health constraint in ruminant livestock production on pasture [1]. Gastrointestinal (GI) nematode infection is a major cause of economic loss in dairy and beef cattle production [1, 2]. Exposure is nearly unavoidable due to grazing stocking rates in non-arid production system environments [3, 4]. Because GI parasite infection can be a serious limitation on ruminant production [5], most producers have undertaken consistent treatment with anthelmintics as a means to control nematode load animals. Continual overuse of these effective compounds has caused development of nematode resistance in some production systems [6]; thus, the emergence of widespread resistance to anthelmintics requires development of new approaches for parasite control [7]. Despite several alternative methods becoming available for infection control including chemical treatment, non-chemical management practices, immune modulation and biological control [8], the high cost of developing new drugs and increasing concerns about their negative impact on the ecosystem requires a need for alternative control approaches based on host response [9]. To minimize the effects of drug residues and yet control economic losses caused by parasitic nematode infections, genetic selection of resistant breeds has been suggested as an alternative control method [10, 11, 12]. Such selection in cattle has not been a primary objective of economic importance relative to genetic improvement of cattle meat and milk production. However, there are examples where producer selection for more resistant sheep has resulted in a steady reduction in flock averages for fecal egg counts (FEC) and drench treatments [13].

Previous studies have suggested that host genetics significantly affects the number of GI nematode eggs per gram (EPG) in the feces of calves, which enables estimation of heritability of resistance [14]. In cattle or sheep, resistance to GI nematodes has been found to be a moderately heritable trait with most heritability estimates approximating 0.3 [1, 15]. Early studies of a closed Angus breeding herd found that EPG counts during the first grazing season were significantly correlated with sire, and that the heritability of this trait was 0.29 [16]. This moderate heritability estimate supports an approach to improve host response to GI parasite infection through marker-assisted selection or genome-wide selection to identify resistant or susceptible individuals for differential management [12, 17].

While the search continues for candidate genes and traits indicative of nematode resistance in ruminants [18], many studies emphasize the relationship between FEC and host genetic differences in an attempt to identify quantitative trait loci (QTL) associated with resistance to GI nematodes [19]. In cattle, two genome-wide significant QTL on BTA 9 and 19 were reported from a microsatellite-based mapping study of 12 paternal half-sib groups made up of 768 Holstein cows [15]. These two QTL corresponded to mapping results from an outcross sheep population, where FEC QTL were found at orthologous positions on OAR 9 and 11 [1]. A more recent bovine mapping study in an experimental Angus herd derived from the Wye farm used 190 microsatellite marker genotypes to identify QTL responsible for the mean FEC variation on BTA 8 and 12 [20].

Because the founder animals of the experimental Angus herd analyzed in both the aforementioned study [20] and this current study were selected for a specific *DRB1* allele to homogenize any potential effects derived from the major histocompatibility complex (MHC) locus, it was important to discern how stratification within this population might influence a genome-wide association study (GWAS). Due to the closed breeding scheme where pedigree coefficients range from 0.1 to 0.35, the genomic autozygosity of this herd should also have increased rapidly. The genomic inbreeding coefficient (*F*) and autozygosity of specific regions can now be calculated directly with the availability of relatively dense and uniformly spaced SNP markers across the whole genome [21]. In principle, autozygosity of an individual depends on common ancestors born 3–5 generation in the past, and these relationships are commonly found in our experimental herd. Such a pedigree structure provides opportunities to investigate inbreeding effects on fitness related to disease resistance [22]. Previously, nematode burden in a wild population of bighorn sheep was found to decline with increased heterozygosity, particularly at the genes involved in disease resistance [23].

In contrast, positive selection of animals can also change the genomic autozygosity by increasing frequency of haplotypes carrying advantageous alleles for fitness. Statistical tests including measuring the decay of association over distance for particular haplotypes by the extended haplotype homozygosity (EHH) [24] or integrated haplotype homozygosity (iHS) [25] tests have been used to detect long extended haplotypes arising by selection. Even small fitness effects can leave a distinct pattern; therefore, it is possible to identify putative disease resistance loci from genomic regions under recent selection [26]. Typically, the haplotype on which a variant has been subject to selection for disease resistance can be found at a high frequency in the population [27]. In humans, an EHH analysis of selection signatures has successfully identified positive selection for an allele bound to the infectious disease resistance [28]. For instance, an allele associated with resistance to malaria increased in sub-Saharan Africans under natural selection [29], and remains as a selection signature across human populations. The correlation between natural or artificial selection and resistance to infectious disease in cattle has been obscure to date.

Experimental measurements of FEC are a costly and time consuming and require animals to undergo parasitic challenge [4]. Thus, the use of QTL associated markers as a predictive measure of FEC should be an essential approach for breeding applications aimed to improve host resistance to GI nematode infection. Furthermore, genomic approaches will present fundamental understanding of gene pathways and allelic variants within gene pathways underlying parasitic disease in livestock. In Angus, initial discovery of QTL controlling FEC using 190 microsatellite markers [20] provided support for further genomic investigations using higher resolution SNP-based marker panels. Such results could provide a more accurate and complete set of selection markers for host responses that control GI parasite infection. Hence, we expanded the parasitic challenge records of our experimental Angus population from 300 to 487 animals, and used dense genome-wide SNP genotypes to more comprehensively locate genomic regions conferring host resistance to GI parasite infection. Positional candidate genes derived from GWAS were then examined using gene annotation tools in an attempt to elucidate the immunological and pathological pathways potentially participating in host resistance differences. In addition, genome-wide calculations of autozygosity (ROH) and selection signatures (iHS) were determined to assess whether inbreeding or positive selection had an impact on resistance to GI nematodes in this Angus population.

## Materials and Methods

### Animals

A selection program for parasite resistance was initiated at the United States Department of Agriculture (USDA), Agricultural Research Service (ARS), Beltsville Agricultural Research Center (BARC) with parental stock derived from the Wye Angus herd at a University of Maryland farm (Queenstown, Maryland). The experiments in this animal study were done under protocol number 05–013 approved by the USDA, ARS, Beltsville Area Animal Use and Care Committee. The pedigree is characterized by 45 half-sib families (n≥5, range 5–27) that originate from two Wye Angus sires born during the 1960s, which are both paternally related to a bull born in 1944. During development of this experimental herd, new sires were from subsequent contemporary groups with EPG phenotype data and were selected to produce calves of desired EPG phenotypes based on this phenotypic data. Animals produced after 1993 were mated to be homozygous at *DRB1* of the MHC locus to homogenize any effects on EPG. The specific haplotype of *DRB1* selected has been found in all *Bos taurus* breeds and present in animals with varying FEC levels. Animals used for GWAS included 487 BARC Angus progeny that underwent parasite challenge studies, which is about a 50% increase in animals compared to our previous study [20]. DNA for genetic analysis from this resource population was acquired from blood and semen. To assess genomic identity between all individuals in the extended families, 99 founding animals and their ancestors (born before 1992) with no parasite challenge records were also genotyped, and DNA was extracted using semen donated by the Wye farm [20].

### Traits

Calves were maintained with their dams on pastures with extremely low numbers of parasites prior to weaning. When the median age of the contemporary group was 205 days, calves were weaned in dry lots for 14 days prior to being placed on pastures naturally contaminated with the two most common nematode parasites in US cattle, *Ostertagia ostertagi* and *Cooperia oncophora*. Calves were monitored weekly for a number of parasitological and immunologic parameters along with selected measurements of animal growth (data not shown). FEC was determined in units of EPG of feces once a week for each animal. Animals born from 1992 to 2007 were included and data collection ranged from 4 to 26 weeks in 17 contemporary groups.

In order to reduce skewness and kurtosis, Box-Cox transformation was applied to raw FEC data for each animal in the challenge experiment, resulting in 487 records of transformed FEC. Transformed values of mean FEC (BC-MFEC) were obtained by analyzing the response variable *y*_*i*_ on a $y_{i}^{\lambda }$ scale obtained from the Box-Cox transformation family [30], in which $y_{i}^{\lambda }=(y^{\lambda }-1)/\lambda ,(\lambda \neq 0)$ or $y_{i}^{\lambda }=\log(y_{i}),(\lambda =0)$. An estimate of lambda calculated from the average FEC measured from the 5^th^ to 26^th^ week was found to be 0.155. FEC was adjusted by the general linear model; y = c + s + βx + e, in which **y** is an individual phenotype (BC-MFEC), c is a contemporary group of an individual, s is sex of an individual, β is the covariate coefficient of age, x is age of animal, and e is the residuals. Birth year of animal (x) and contemporary group (c) effect were included in the statistical model to correct for any contemporary group effects. Using the same model above, the heritability (*h* ^2^) of BC-MFEC was estimated using a mixed model including contemporary group, sex, animal age, an individual animal relationship, and random error effects. Serum pepsinogen levels were measured as described previously and log-transformed to normalize their values [14].

### GWAS

A total of 586 Angus produced between 1964 and 2007 were genotyped using the Illumina BovineSNP50 (San Diego, CA) according to the manufacturer’s protocol. Illumina’s Genome Studio was used to determine 45,632 SNP genotypes with an average call rate over 99%. Genome coordinates of each SNP were determined according to UMD 3.1 genome assembly [31, 32]. SNP marker genotypes with a MAF <0.05 or failing the Hardy-Weinberg equilibrium test (Fisher’s exact test, *p*<0.0001) were discarded. Any SNPs with <50% call rate across the population were also excluded. A total of 31,165 SNPs were retained for a single marker-trait association tests. For haplotype analysis, SNP marker genotypes were increased to 39,273 by lowering MAF (<0.02) thresholds in an attempt to better identify regions under the influence of inbreeding and/or positive selection. DAVID analysis was used in an attempt to elucidate common function among the candidate genes residing within genomic regions containing suggestive and significant BC-MFEC SNP associations [33]. Annotation of these genes was also checked manually using the KEGG database [34], InnateDB [35], and the UCSC browser [36, 37].

Association between SNP and BC-MFEC was evaluated with a linear mixed model including the effects of SNP genotype and a polygenic effect. A regression analysis was performed to detect the additive genetic effect of SNP across the genome. The association between haplotype and adjusted data was evaluated using a generalized linear model, y = μ+ βG + u + e, where y is the vector of residuals of BC-MFEC of individuals adjusted for sex, age, and contemporary group, μ is mean, and β is a vector of additive genetic effect. The adjusted value (y) is used as independent variable in the mixed model. G is an indicator variable for the additive genetic effect of an individual, and *e* is vector of individual error terms. Then u is a polygenic (random) effect using individual relationships, which was assumed to be distributed u~(0, Aσ_u_^2^), where A is the additive individual relationship among animals based on pedigree. R with the kinship package [38] was used for the analysis of association including polygenic effect in a mixed model. Statistical thresholds were determined accounting for multiple comparisons. To decide the threshold level for a genome-wide scan, a permutation test was used [39]. The genome-wide significance of the significance was determined by comparison with the distribution of the largest genome-wide test statistics obtained from the analysis of 1,000 permutations (adjusted *p* = 0.01; 3.0 x 10^-5^).

In addition to the above GWAS, GenSel [40] was used to estimate the variation explained by the regions associated with BC-MFEC. The Bayes B approach with π = 0.995, where π is the prior proportion of SNPs assumed to affect no genetic variation on mean FEC, with a Markov chain of 50,000 iterations. BC-MFEC was used as the trait. The posterior samples of the effects of all SNPs were summarized using a 1-Mb window in the candidate regions defined based on results of our GWAS.

### Signatures of selection

To examine selection for a specific *DRB1* haplotype of the MHC locus or any traits corresponding to regions associated with resistance to GI nematodes, we determined signatures of selection using an EHH analysis. Haplotypes were determined using Fastphase [41], and evidence for positive selection was calculated from the value of the standardized integrated extended haplotype score (iHS) for each SNP [25]. This test evaluates relative decay of EHH of ancestral and derived core alleles. Because of high levels of autozygosity in our Angus herd, we scanned extended haplotype boundaries up to 10 Mb from the core position, and ancestral type alleles were derived using BovineSNP50 genotypes from the Bovine HapMap genotype panel [42]. Raw iHS was standardized across the whole genome except for the sex chromosomes, in order to obtain the unit of standard deviation with a mean of 0 and variance of 1. The large absolute value of standardized iHS (>3) is expected to represent haplotypes selected by recent, strong selection of a gene variant associated with the desired phenotype. The modified C++ source code from the developers of iHS [25] and R [38] were used to calculate the standardized integrated haplotype score statistic t.

### Genomic autozygosity and ROH-trait association

Runs of homozygosity (ROH) were calculated across the autosomes to investigate the effect of autozygosity relative to the marker-trait association results. ROH was defined by stretches of continuous homozygous SNPs spanning at least 1.5 Mb with 100 or more SNPs, which enables calculation of local autozygosity most likely derived from recent common ancestors. The threshold for ROH was decided based on the expected length of an autozygous ROH segment (0.5 Mb with 50 SNPs) caused by a common ancestor born 30 generations ago [43]. Next, genomic autozygosity of a locus was calculated using the sum of ROH status for each SNP in all individuals divided by the total number of animals.

In order to ascertain the effect of local autozygosity on BC-MFEC, the associations between ROH and GWAS results for each animal were analyzed using a linear regression [44]. In our model, BC-MFEC was used as a response and ROH status of a SNP (0 or 1) was included to examine the effect of autozygosity on this trait. It was possible this approach would reveal the influence of recessive deleterious mutations originating from influential ancestors. Additionally, we compared the level of ROH between ancestral animals produced before 1990 (N = 70) and contemporary animals born after 2004 (N = 85), which could represent a change of local autozygosity due to mating between related animals in the pedigree. Detection of ROH and statistical tests were performed using Perl and R.

## Results

### FEC and marker associations

Initially, the heritability of GI nematode burden was estimated in 487 Angus animals. The original fecal egg count was zero-skewed data; therefore, the data was Box-Cox transformed (BC) to ensure the validity of normal distribution and related assumptions [45]. The Shapiro-Wilk test indeed proved the normality of BC-MFEC (*p*<0.05). Using a mixed model, the narrow-sense heritability of BC-MFEC was estimated at *h* ^*2*^ = 0.42, which was higher than estimated *h* ^*2*^ of GI nematode burden in previous cattle studies [12, 15]. While the effect of animal age or sex of an individual was not significant, effect of contemporary group affected variation in BC-MFEC significantly (*p*<0.0001).

**Fig 1:**
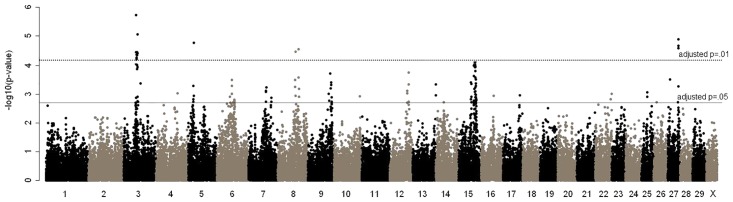
Manhattan plot of BovineSNP50 marker association tests with transformed mean FEC. Each dot indicates significance level (–log_10_p) of an association for BovineSNP50 marker relative to its UMD 3.1 genome coordinate. Suggestive and significance thresholds are described in the Materials and Methods.

To detect genomic regions affecting traits related to GI nematode burden in Angus, a GWAS was done using the residuals of BC-MFEC (Fig. 1). The average distance between informative markers was approximately 100 kb, and a total of 104 suggestive and significant SNP marker:BC-MFEC associations (adjusted *p*≤0.05) were detected on 19 chromosomes. The majority of these associations (N = 69) were located on BTA 3, 6, 9, and 15 (Table 1). Only 13 associations reached a significance threshold at the adjusted *p*≤0.01 (minimum equivalent to-*log*_*10*_ *p* = 4.3), and these markers were located in six regions on BTA 3, 5, 8, 15, and 27 (Table 1; S1 Table). The BC-MFEC associations identified using GenSel agreed those found in our initial GWAS considering the broad candidate regions, including the intervals on BTA 3, 6, 9 and 15 (data not show). No significant associations were detected for serum pepsinogen levels.

**Table 1:** Summary of most significant genomic regions associated with transformed mean FEC.

BTA	Region (Mb)^1^	Maximum association^2^	Number of associations^3^	Gene^4^	Variation (%)^5^
3	51.94–62.01	52.63 (5.72**)	19	*BARHL2*	2.68
5	19.02–21.60	21.60 (4.77**)	6	*BTG1*	0.35
6	48.67–66.21	55.21 (3.30*)	10	*FOXP1* ^*6*^	2.57
7	64.19–66.89	66.89 (3.25*)	4	*GRIA1/ADAL*	0.40
8	65.12–67.50	67.50 (4.40**)	4	*LPL*	0.26
	78.70–80.42	78.70 (4.49**)	4	*NTRK2*	0.30
9	86.21–96.74	91.59 (3.72*)	10	*RGS17/MIR2480*	1.41
12	68.81–75.90	75.67 (3.77*)	6	*CD164* ^*6*^ */ABCC4* ^*6*^	0.67
15	48.65–50.93	49.45 (3.37*)	6	*HB complex*	0.26
	57.83–68.19	65.33 (4.27**)	30	*FBXO3*	2.45
22	58.58–60.27	60.12 (3.02*)	3	*GATA2*	0.21
27	37.76–39.06	39.02 (4.89**)	5	*NAT1*	0.70

^1^UMD 3.1 genome coordinates containing multiple suggestive associations.

^2-^log_10_p value,

*suggestive,

** significant.

^3^Number of associations that surpass suggestive level (*p*<0.05).

^4^Gene located nearest to the maximum association based on UCSC browser of UMD 3.1 [36, 37].

^5^Percent of phenotypic variation as determined by GenSel [40].

^6^No annotated bovine reference genes, annotation from bovine EST data or human reference gene alignment in UCSC browser.

Based on the GWAS results, we next focused on regions with a large number of localized SNP associations for BC-MFEC. In total, 12 regions encompassing multiple associations were contributing to significant variation in this trait (S1 Fig.). The genetic variation explained by SNPs affecting BC-MFEC did not exceed 3% for any candidate region and totaled about 13% across all 12 candidate regions (Table 1). A broad region (51.9–62.0 Mb) on BTA 3 comprised 19 suggestive or significant single marker associations including the most significant associations (52.6 Mb) across the genome. Similarly, 30 SNP located within 58–69 Mb of BTA 15 suggested existence of allelic variants contributing to GI nematode resistance in extended families.

Approximately 100 annotated genes related to immune responses or susceptibility to disease were identified from the 12 genomic regions associated with BC-MFEC (Table 1). Analysis of the annotation from this gene set revealed prevalence for immunological pathways participating in chemokine signaling, leukocyte migration, and hematopoietic stem cell development (Table 2). This cursory survey of positional candidate genes supports the hypothesis that genetic differences in the innate and/or adaptive immune response affect resistance to nematodes in our population.

**Table 2:** Positional candidate genes related to immunological pathways.

Pathway	Genes^1^	Function^2^
Toll-like receptor signaling pathway	*TLR1* (6), *TLR6* (6), *TRAF6* (15)	Chemotactic effects (NK cell, T cell), T cell stimulation, Antiviral effects, Proinflammatory effects
Chemokine signaling pathway	*ULBP1* (9), *PRKACB* (3), *CCL19/21/27* (8)	Leukocyte transendothelial migration
Leukocyte transendothelial migration	*RhoH* (6), *Nox3* (9), *EZR* (9), *CLDN10* (12)	Inflammation, immune surveillance
Hematopoietic cell lineage	*CD44* (15), *GP9* (22)	Differentiation of hematopoietic stem cell into white blood cells, leukocytes-the natural killer (NK) cells, the T and B lymphocytes
Cytokine-cytokine receptor interaction	*CNTFR* (8), *KITLG* (5)	Intercellular regulators and mobilizers of cells engaged in innate and adaptive inflammatory host defenses

^1^Gene ID (Chromosomal location).

^2^Annotation in KEGG pathway [34].

### Signatures of selection

**Fig 2:**
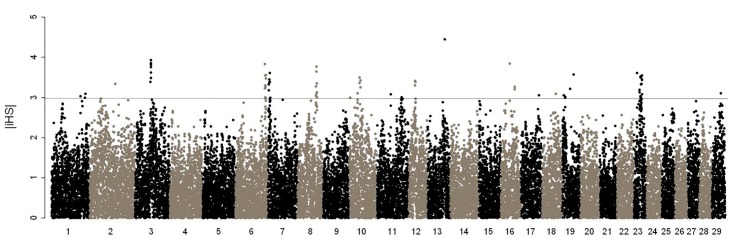
Genome-wide plot of standardized |iHS|. Each dot represents |iHS| of a SNP that is located at the center of an extended haplotype (10 Mb) plotted against its genome coordinate on UMD 3.1. Dotted line is |iHS| = 3.

Signature of selection analysis detected 71 loci with |iHS|>3 (Fig. 2). To reduce the number of potential false positives, these regions were summarized by embracing haplotype core SNPs with |iHS|>3 flanked by multiple |iHS|>2 scores (Table 3). As expected, substantial evidence of selection was identified in the MHC region (26–31 Mb) on BTA 23 (S2 Fig.). A second region apparently under considerable selection encompassing seven signals with |iHS|>3 was found in a 2.1 Mb fragment on BTA 3 (62.57–64.69 Mb). Five additional regions were also found that may represent recent signatures from founding animals derived from closed herd breeding at the Wye Angus herd or from differences with the Angus breed.

**Table 3:** Genomic regions encompassing significant |iHS| scores.

BTA	Region (Mb)^1^	Maximum |iHS|	Position (Mb) ^2^	Significant |iHS|^3^
3	62.57–64.69	3.93	63.11	7
6	106.08–108.83	3.83	106.32	10
7	3.55–6.47	3.62	6.47	6
8	86.34–90.26	3.77	88.81	8
10	29.51–43.49	3.50	40.58	5
12	33.11–35.23	3.41	33.11	3
23	23.74–31.52	3.56	31.10	15

^1^ Region represents location between the first and the last core SNP with |iHS|>3.

^2^ The UMD 3.1 genome coordinate of SNP with the highest |iHS| score in the designated signature region.

^3^ The number of core haplotype SNPs with |iHS|>3 in the designated signature region.

Although the genomic regions under selection were sometimes located nearby regions associated with parasitic disease resistance (e.g., BTA 3 and 8), no signature regions entirely overlapped with our GWAS results. Interestingly, two markers associated with mean FEC (62 and 64 Mb) were also found to be under moderate levels of selection (|iHS|>2) on BTA 15 (S1 Table). Collectively, no single core SNP within a haplotype and associated with BC-MFEC (genome-wide association, adjusted *p*≤0.01) appeared to be under significant positive selection with |iHS|>3.

### Runs of homozygosity

**Fig 3:**
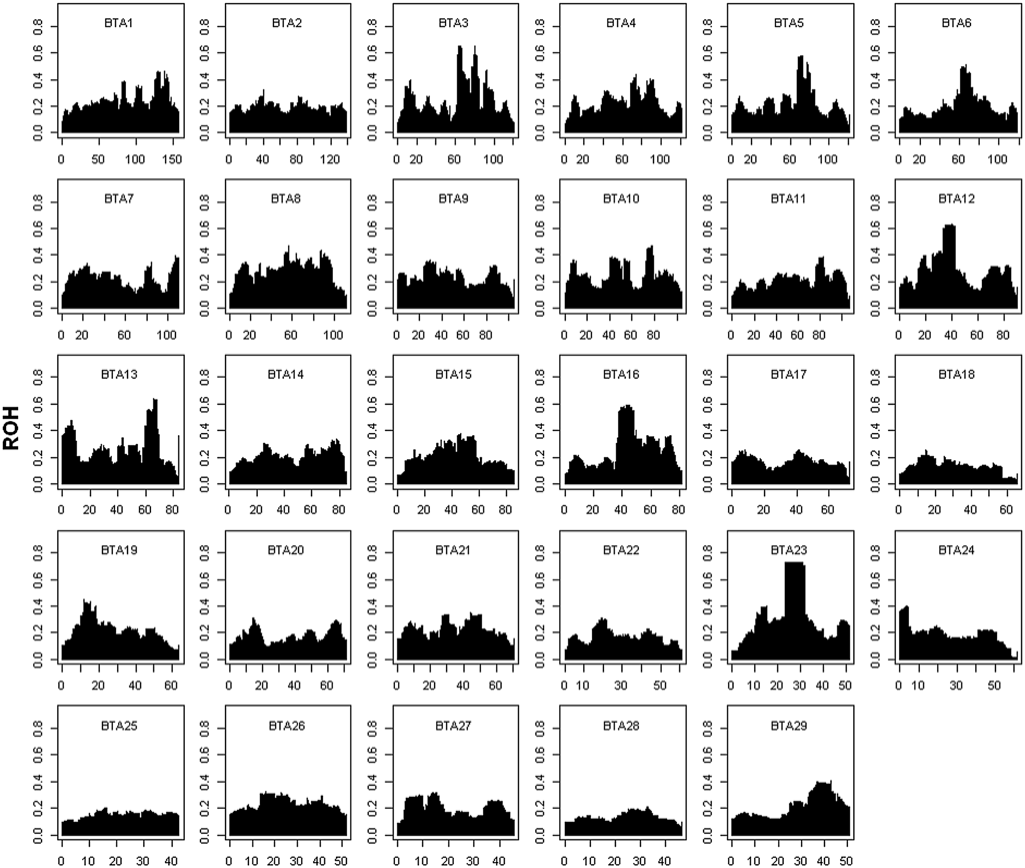
Runs of homozygosity (ROH) in each chromosome. The levels of ROH (y-axis) are plotted against the SNP genome coordinate on UMD 3.1 (x-axis).

To identify potential correlations between autozygosity and resistance to disease, the genomic homozygosity was measured using ROH detected across sampled Angus genomes with a threshold set at 1.5 Mb with 100 or more SNPs. The typical size of a homozygous genomic region ranged between 2–5 Mb, and the longest haplotype homozygosity of an individual extended over 50 Mb encompassing almost half of BTA 7. The average autozygosity of a marker locus defined by ROH was 0.21, varying from 0.02 to 0.73 (Fig. 3; S3 Fig.). In all, autozygosity surpassing 0.4 was detected for 12 chromosomal regions (Table 4), which was equivalent to top 2% of ROH levels across all genomes. Only two out of 15 ROH regions with high homozygosity (>0.4) contained SNP with suggestive associations to BC-MFEC. One of these was just distal to the significant genome region found on BTA3 (Table 1), while the other partially overlapped the suggestive region on BTA 6. Comparing the other candidate genomic regions determined by GWAS, six suggestive regions had moderate autozygosity (0.2–0.4) and the three significant regions on BTA 3 (51–62 Mb), BTA 15 (58–68 Mb), and BTA 7 (64–66 Mb) had relatively low autozygosity (0.13–0.17).

**Table 4:** Summary of genomic regions with ROH>0.4.

BTA	Region^1^	Maximum ROH	SNP Associations^2^	Change of ROH^3^
1	126.71–141.77	0.46	-	+0.15
3	62.11–92.29	0.65	1*	+0.27
4	71.64–74.27	0.44	-	+0.13
5	67.54–80.79	0.58	-	-0.13
6	60.72–71.36	0.51	4*	+0.11
8	52.72–55.92	0.42	-	-0.08
	86.73–90.62	0.43	-	+0.19
10	72.88–79.93	0.45	-	+0.35
12	25.01–42.51	0.62	-	+0.24
13	2.07–9.66	0.46	-	+0.22
	59.22–69.48	0.64	-	+0.19
16	59.22–68.92	0.59	-	+0.23
19	11.69–15.58	0.43	-	-0.09
23	23.55–31.97	0.73	-	+0.59

^1^Regions representing homozygosity >0.4, where homozygosity was calculated using ROH status of each SNP in a ≥100 SNP window.

^2^Number of SNP associated with BC-MFEC in this genomic region,

* suggestive.

^3^Maximum change of ROH in the region between ancestors born before 1991 and animals born in 2004–7.

### Effect of change in autozygosity on resistance to nematodes

As shown above, the autozygosity of the MHC region has increased greatly from 0.3 to 0.9 (*-log*_*10*_ *p*>10), resulting in high levels of ROH and a strong selection signature on BTA 23 (S4 Fig.). Selective mating between the animals with identical *DRB1* haplotypes during the past 15 years caused this phenomena, whereas the change in autozygosity for other autosomal regions during the same time period ranged from -0.2 to 0.35 (*p*<0.001) (Table 4). In particular, signatures of selection on BTA 3, 8, and 12 (Table 3) appear to be derived from selective breeding or genetic drift during develop of our experimental herd based on changes in ROH relative to the Wye ancestors. From these results, we conclude that the regions associated with BC-MFEC in our experimental Angus population are not obviously correlated with recent artificial selection or inbreeding.

**Fig 4:**
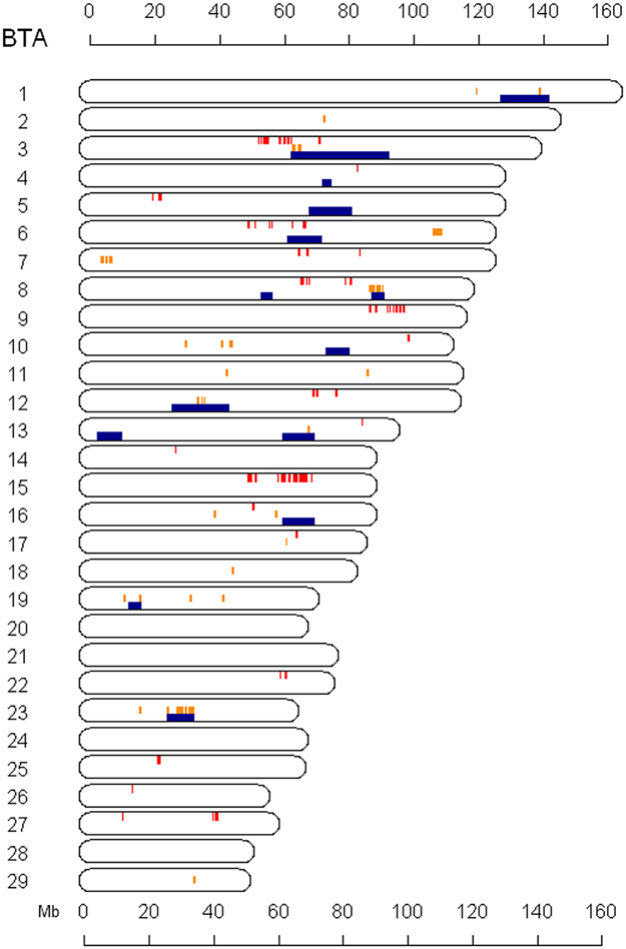
Comparison of genomic locations for GWAS, signature of selection, and ROH results. Each bar in red (upper), orange (middle), and blue (lower) represents SNP:BC-MFEC associations (adjusted p<0.05), |iHS|>3.0, and ROH>0.3, respectively.

To investigate potential effects of local autozygosity across the genome, we examined the genomic co-localization of BC-MFEC GWAS results, selection signatures (|iHS|), and ROH-derived genomic autozygosity (Fig. 4). This plotting across the autosomes showed only BTA 3 and 6 have some evidence of selection for variants affecting BC-MFEC, while BTA 8 showed an unusual pattern of selection in genomic regions flanking variants associated with parasite resistance. We also investigated if there was a recessive mode of inheritance for variants affecting BC-MFEC by regressing ROH for each animal with GWAS results for BC-MFEC (S5 Fig.). Overall, there was a strong signal on BTA 11, but no significant associations between ROH and BC-MFEC were found, while many regions across the genome seem to moderately contribute to mean FEC.

## Discussion

A management program based on DNA tools that help identify resistance and/or susceptibility to GI nematode parasites in cattle has potential to decrease the dependence on anthelmintics, reducing risk of environmental residues and creation of drug-resistant parasites. Availability of a bovine reference genome [31], genome-wide SNP genotyping platforms [42], and phenotyped animals with estimated heritabilities for FEC reaching 0.4 [45] suggested that a GWAS of our experimental Angus population (N = 487) would yield molecular genetic information about host resistance to GI nematode transmission and infection that could be applied to development of DNA tools for selection and management.

Although only a few QTL for GI nematode resistance had been identified in cattle, our study is the first to report genomic locations using a GWAS based on dense genome-wide SNP genotypes. We found 12 genomic regions containing multiple SNP associations, which accounted for about 13% of the variation in FEC in our closed breeding population. We also confirmed the results for our previous microsatellite-based QTL mapping by validating BC-MFEC associations on BTA 8 and 12 [20]. We still found that none our associations were similar to those QTL on BTA 9 and 19 identified in Holsteins [15]. The discrete genomic region we detected on BTA 9 containing 10 suggestive SNP is just distal to a broad QTL region on BTA 9 previously described [15]. It is possible that several differences between the two studies including breed, pedigree structure, parasite challenge protocol, and age of measurement of FEC may have caused differences in location between association results. In our case, FEC measures were derived from a natural challenge of presumed naïve animals within a limited age range (shortly after weaning), while the other study determined numbers of nematode EPG for cows of various ages in a commercial dairy setting [15].

To provide some additional support, we compared our GWAS findings to those found in three recent reports using genome-wide SNP-based methods to detect genomic regions affecting host resistance to GI nematodes in sheep [46–48]. In a signatures of selection study comparing two lines of purebred sheep divergently selected for susceptibility and resistance to GI nematodes, 16 genomic regions under divergent selection were identified, and two of these regions corresponded to previously detected sheep QTL for FEC [46]. We identified two common regions on OAR 1 and 19, which were comparable with our findings on BTA 3 and 22, respectively (Table 1). Interestingly, the narrowly defined regions on both OAR 1 and BTA 3 completely overlap and contain only one annotated gene, *BarH-like homeobox 2* (*BARHL2*). This gene encodes a transcription factor involved in cell fate commitment and neuron differentiation; however, there is no bovine gene expression data to provide clues as to the tissue expression pattern of *BARHL2* [35]. Although this comparison is compelling, the genomic structure of our animals (relatively high genomic autozygosity and complex pedigree relationships both extending LD and IBD) may preclude only looking at genes within discrete genomic regions. Therefore, we also identified that *Growth factor independent 1 transcription repressor* (*GFI1*), which is a transcription repressor induced by T cell activation and IL-4/Stat6 signaling that enhances Th2 cell expansion [49], is located nearby at 55 Mb on BTA 3. The comparable regions on OAR 19 and BTA 22 are located 4 Mb apart in gene rich regions for both genomes, so no positional candidate genes could be easily identified. The closest gene in our study is *ABCC4*, which is implicated in multi-drug resistance and platelet degranulation [35]. Comparison of our GWAS results to the two other GWAS results in sheep [47, 48] found no overlap between cattle and sheep genome coordinates of FEC-associated markers. It should be noted that the study by Salle and colleagues [48] did detect a QTL for pepsinogen levels on OAR 21, which corresponds to BTA 29 (39.05 Mb).

In our challenge studies, truly resistant animals shed very few worms based on raw FEC while animals with a continually increasing FEC up to the 26^th^ week are categorized as susceptible possibly due to an improper immune response to infection [12]. Cases where initial FEC spikes and then decreases post-infection are presumed to be typical of animals mounting an adaptive immune response to infection. About 50% of our phenotyped animals belong to this latter class of immune response; whereby resistance to subsequent parasitic infection is gained through adaptive immunity. However, when trait measures for FEC during the last 4 weeks were tested no associations (adjusted *p*≤0.05) were detected to distinguish susceptible animals from innate and adaptive resistant animals (data not shown). We considered this an unfortunate result, because selection against susceptible animals may be the most effective disease control strategy for parasite infection. Like sheep [50], the highly susceptible cattle largely contaminate the pasture to perpetuate parasite infection of the herd.

There are a number of characteristics for our population, which may have hindered detection of QTL for parasite indicator traits. First, a previous pedigree analysis of our population revealed more than 90% of the animals are paternally inherited from a Wye bull born 70 years ago [20]. This population structure is likely to be useful when beneficial alleles have segregated to descendants, facilitating detection of marker-trait association. In contrast, the possibility of detecting newly introduced disease resistance alleles is small due to the closed nature of the pedigree and pedigree loops prevalent in the animals of our herd. Furthermore, complex inbreeding loops have been an obstacle to estimate identical by descent (IBD) haplotypes of distantly related individuals in the extended family. The largest half-sib family consisted of 18 animals and only 5 families have more than 10 offspring, whereas about 80% of the animals are members of small half-sib families (N≤10), reducing power to detect QTL using linkage mapping.

Furthermore, the selection for a single *DRB1* allele in our population may also have diminished our ability to detect marker:FEC associations across the genome. The closed mating system substantially reduced the amount of variation across the entire genome, where we observed that more than 20% of the whole region consists of long haplotypes (>1 Mb) at high frequency (>0.5) based on 50 or 100-SNP windows. As a consequence of increasing genomic homogeneity in our closed herd, reduced polymorphism may have decreased our power to detect associations with parasite-related traits.

Haplotype-based association tests have also been suggested [51] as an alternative to single marker methods, which require no prior knowledge of haplotypes and IBD to improve mapping accuracy as available markers increase [52]. According to linkage disequilibrium (LD) calculations reported for dairy cattle, 300,000 or more evenly distributed SNPs may be necessary for a high-resolution genome-wide scan, while 50,000 SNPs would be sufficient for association studies in a highly inbred population [53]. As stated previously, long range haplotypes (>1 Mb), which originate from recent common ancestors in the closed Wye Angus herd have possibly hindered narrowing of GWAS results. Recent advances in sequencing technology could provide enough markers (e.g. 0.7 million SNPs) to cover the entire genome, decreasing the need for an association test based on haplotypes or markers in LD. Using simulated data with high LD, a comparative study of the power and precision of several methods for LD mapping concluded that single-marker regression was equal or superior to other regression methods and comparable to LD mapping using haplotypes and IBD probabilities [54].

Due to the above characteristics of our experimental herd, we attempted to exploit signature of selection and genomic autozygosity analyses as alternatives to find potential genomic regions under selection and affecting parasite infection. Positive selection can increase the rate at which deleterious mutations accumulate directly, when the effect of the advantageous mutation outweighs the effects of linked deleterious mutations [55], or indirectly, through a reduction in effective population size mediated by an increase in the variance of reproductive success [56]. There is also evidence suggesting ROH represents recent artificial selection for economic traits in dairy cattle [57]. In our current study, genome-wide ROH analysis identified 14 substantial regions with high ROH >0.4 (Table 4), but only two regions (BTA 3 and 6) showed nominal evidence of potential relationships between genomic autozygosity and marker associations with BC-MFEC. Interestingly, two genes within this shared region on BTA 6 are *TLR1* and *TLR6*. These genes belong to a class of genes found to be highly expressed in GI nematode resistant sheep [58]. Recent studies in humans and mice also suggest that genetic variation of immune related traits in mammals may be caused by polymorphisms in genes that encode important proteins in Toll-like receptor signaling pathways [59]. Overall, we found only two genomic regions on BTA 3 and 10 showing considerable change of ROH (>0.25) during the last 20 years outside of the obvious near fixation at the selected MHC region on BTA 23. This result implies that the influence of local autozygosity on parasite resistance did not change substantially over the past 20 years of closed breeding. Furthermore, the analysis of association of ROH and BC-MFEC shows little support for genomic regions with high autozygosity affecting host resistance to GI nematode infection (S5 Fig.).

For the signature of selection analysis, we could only detect moderate or weak positive selection for loci associated with FEC, which suggests the methods used for selective mating between animals with similar values of FEC were not effective. For example, environmental effect explained ~60% of total variation of FEC, but the selection of animals with high or low FEC was not dependent upon a fully adjusted genetic value, which likely decreased the accuracy of selection. The standardized value of iHS is optimized for detecting evidence of allelic selection with frequency 0.7–0.9 [25], but this was not the case in our study. In addition, detection of selection or association with a trait is almost impossible when the favorable allele is nearly fixed (e.g. frequency >0.95). Also, any detection of selection signatures could reflect artificial selection of other traits in the Angus breed prior to the 1960s or the Wye farm since the 1930s. The selection signatures could be interpreted as an evidence of resistance to infectious disease that might affect the frequency of genes involved in immune response (S2 Table). Although we could not clarify the causes of other regions with significant values of iHS, our evidence considering the specificity of iHS for the detection of selection signatures in the MHC region was considerably strong (S4 Fig.).

In sheep, comparison of gene expression differences between genetically resistant or susceptible lines has also been used as a method to identify genes that contribute to an effective immune response against GI nematodes [58]. Even without gene expression data, this approach may be useful in our population, if disease response is accurately characterized through annotation of positional candidate genes found by GWAS. The DAVID analysis provided useful insights into the potential biological mechanism underlying GI nematode resistance in cattle. In comparison to previous expression studies from our population, genes encoding mediators of the inflammatory response exhibited an elevated expression of inflammatory cytokines such as *TNFα*, *IL-1β*, and *MIP-1α* in fundic and pyloric abomasa 7 days post infection [60]. These findings indicated resistant animals maintained inflammatory responses at the site of infection. During the inflammatory response, transendothelial migration of leukocytes involves a spatio-temporal regulation of adhesion molecules, chemokines and cytoskeletal regulators [61]. Distinct steps of leukocyte transendothelial migration are regulated by sequential integrin activation and coordinated Rho family GTPase activity [61], some of which are affected by disease resistance. Our annotation results of candidate BC-MFEC genomic regions were in agreement that genes influencing leukocyte activation, migration and functions are important to the resistance response to infection; however, these positional candidate genes were not detected by previous differential expression studies of cytokines in resistant or susceptible animals from our herd [60].

Availability of bovine genome-wide SNP genotyping platforms has been and will continue to be a useful tool for developing selection programs in cattle [62]. However, resistance to parasitic disease has not been considered the primary aim of selection for any commercial cattle breeding programs. Our genomic analyses suggest there are many genomic regions contributing to the resistance or susceptibility of GI parasite infection in an animal, which supports previous findings in sheep that genome-wide selection methods may be the best option for rapid genetic improvement of host resistance [17]. It also underscores the need for additional GWAS in other cattle breeds and production systems to develop genome selection systems for parasite traits. Since genes associated with disease resistance have not been under apparent artificial selection, the effect of selection for host resistance alleles would be expected to be substantial. On the other hand, identification of resistant or susceptible animals for research purposes could enhance knowledge of genes and their variants that make up the immune responses involved in GI nematode infection of ruminants [9]. Certainly, genomic regions associated with GI disease susceptibility need to be studied further, since removing animals causing the most disease transmission is probably the best first step towards developing efficient and sustainable control programs for parasite infection.

## Supporting Information

S1 Fig Chromosome plots of GWAS results for BC-MFEC. (TIF)

S2 Fig Chromosome plots of |iHS| scores of core SNPs from Fastphase haplotypes. (TIF)

S3 Fig Genome-wide plot of runs of homozygosity. (TIF)

S4 Fig Signature of selection |iHS| and genomic autozygosity (ROH) at MHC locus on BTA 23. (TIF)

S5 Fig Genome plot of association test statistics between ROH and BC-MFEC. (TIF)

S1 File Contains S Fig. Legends, SNP map, Genotype, Phenotype, and Pedigree files. (ZIP)

S1 Table Summary of all significant BovineSNP50 markers associated with BC-MFEC. (DOCX)

S2 Table Genes related to immune response in genomic regions under selection (|iHS|>3). (DOCX)
