# Supplementary figures and images for: Genome-Wide Scan of Gastrointestinal Nematode Resistance in Closed Angus Population Selected for Minimized Influence of MHC
Source: PLoS One. 2015 Mar 24;10(3):e0119380. doi: 10.1371/journal.pone.0119380 (PMC4372334; doi:10.1371/journal.pone.0119380)

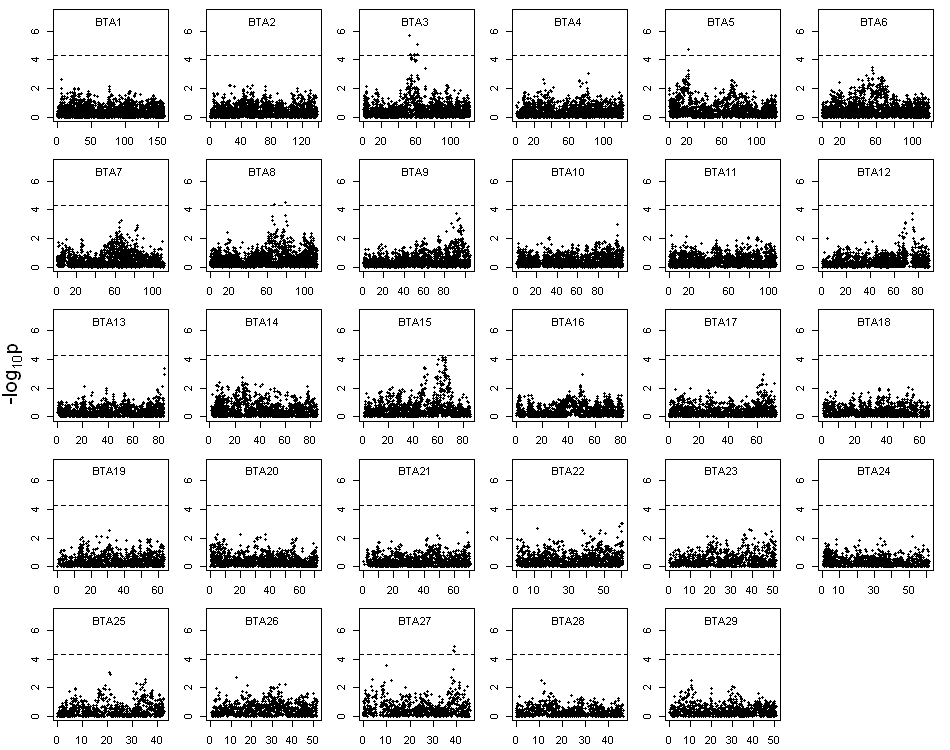

Supplement: S1 Fig — (TIF) [file pone.0119380.s001.tif]

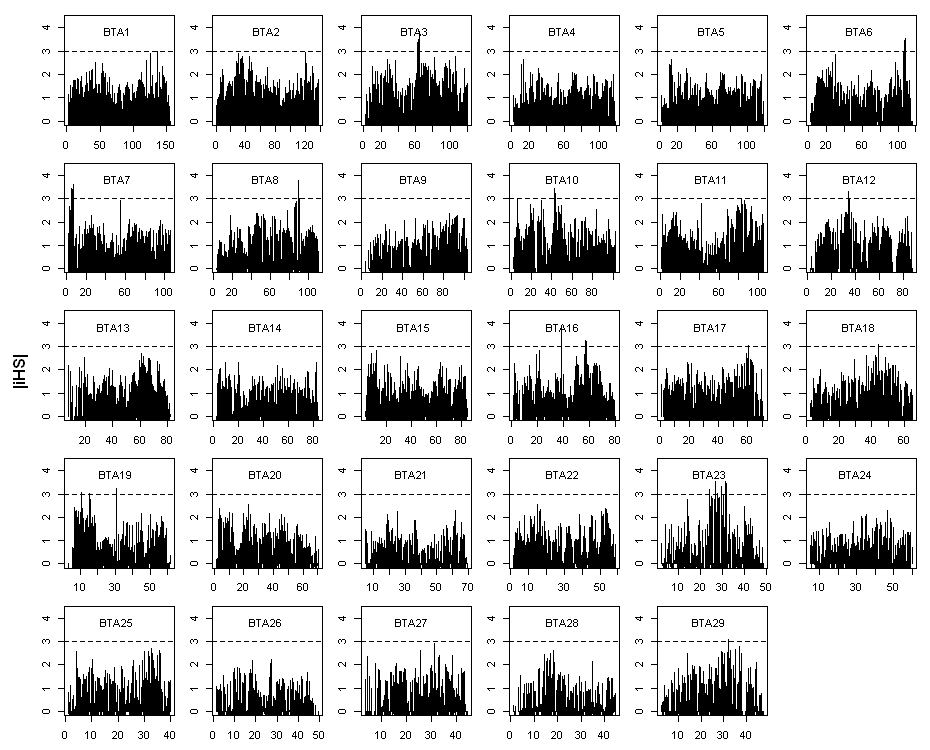

Supplement: S2 Fig — (TIF) [file pone.0119380.s002.tif]

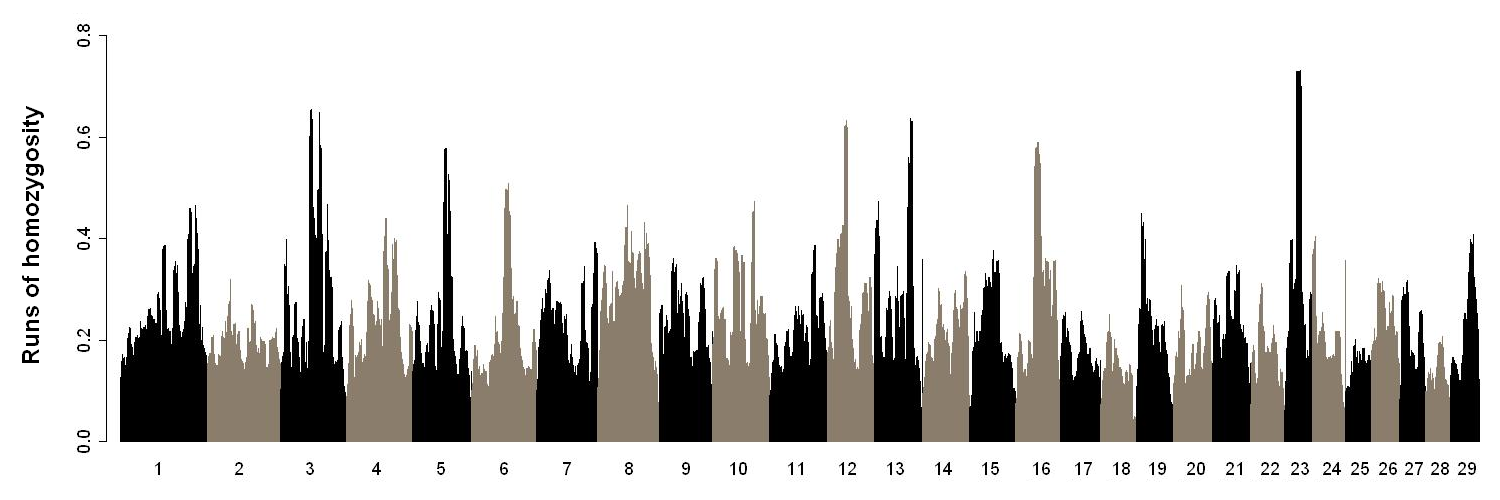

Supplement: S3 Fig — (TIF) [file pone.0119380.s003.tif]

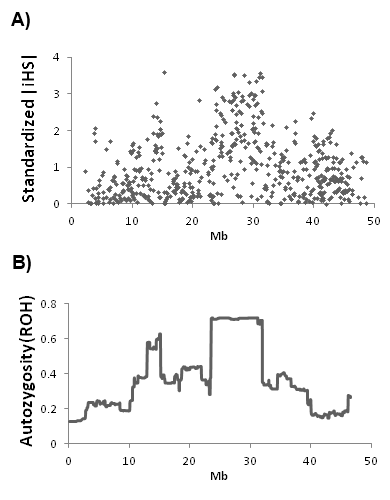

Supplement: S4 Fig — (TIF) [file pone.0119380.s004.tif]

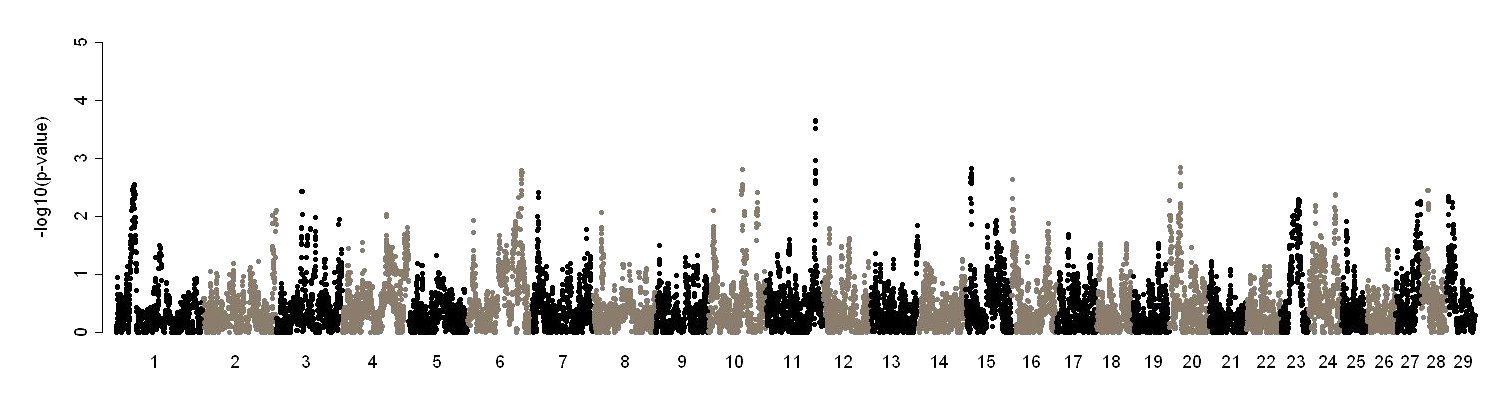

Supplement: S5 Fig — (TIF) [file pone.0119380.s005.tif]
