# Supplementary material for: Genome-Wide Scan of Gastrointestinal Nematode Resistance in Closed Angus Population Selected for Minimized Influence of MHC
Source: PLoS One. 2015 Mar 24;10(3):e0119380. doi: 10.1371/journal.pone.0119380 (PMC4372334; doi:10.1371/journal.pone.0119380)
Supplement: S1 File — (ZIP) [file pone.0119380.s006.zip › SI File/S Fig. Legends.docx]

**S1 Fig. Chromosome plots of GWAS results for BC-MFEC.**

Each box is a chromosomal plot based on genome coordinates from UMD 3.1. Each dot indicates significance level (–log_10_p) of an association between SNP genotypes and BC-MFEC.

**S2 Fig. Chromosome plots of |iHS|** **scores of core SNPs from Fastphase haplotypes.**

Each box is a chromosomal plot based on genome coordinates from UMD 3.1. Each plotted bar represents the |iHS| score of a core SNP that is located at the center of the extended haplotype (10 Mb). Dotted line is |iHS|=3.

**S3 Fig. Genome-wide plot of runs of homozygosity.**

The levels of ROH (y-axis) are plotted against the UMD 3.1 genome coordinates (x-axis) of a SNP. Chromosome numbers are shown on x-axis.

**S4 Fig. Signature of selection |iHS| and genomic autozygosity (ROH) at MHC locus on BTA 23.**

Each dot represents a SNP marker position plotted against the iHS score (A) and ROH of each locus is displayed with a connected line (B).

**S5 Fig. Genome plot of association test statistics between ROH and BC-MFEC.**

Significance levels of association between ROH and BC-MFEC are plotted across the genome. Chromosome number is shown on x-axis.
