# Supplementary material for: Genome-Wide Scan of Gastrointestinal Nematode Resistance in Closed Angus Population Selected for Minimized Influence of MHC
Source: PLoS One. 2015 Mar 24;10(3):e0119380. doi: 10.1371/journal.pone.0119380 (PMC4372334; doi:10.1371/journal.pone.0119380)
Supplement: S1 Table — (DOCX) [file pone.0119380.s007.docx]

**S1 Table. Summary of all significant BovineSNP50 markers associated with BC-MFEC.**

| **BTA** | **Marker** | **Position (Mb)** | **-log_10_p** | **\|iHS\|^*^** | **MAF** |
| --- | --- | --- | --- | --- | --- |
| 3 | Hapmap59681-rs29019879 | 51942884 | 2.718 | 0.59 | 0.29 |
| 3 | Hapmap60328-rs29027404 | 52631451 | 5.721 | 0.49 | 0.27 |
| 3 | Hapmap43965-BTA-89883 | 53548981 | 4.381 | 0.05 | 0.22 |
| 3 | Hapmap54072-rs29012393 | 53831533 | 2.707 | 1.21 | 0.33 |
| 3 | Hapmap48848-BTA-67904 | 54236911 | 4.063 | - | 0.16 |
| 3 | ARS-BFGL-NGS-43853 | 54285794 | 2.888 | - | 0.28 |
| 3 | ARS-BFGL-NGS-12447 | 54466460 | 4.295 | - | 0.18 |
| 3 | BFGL-NGS-117514 | 54573707 | 4.138 | 0.18 | 0.21 |
| 3 | BTA-92147-no-rs | 54680882 | 4.201 | - | 0.18 |
| 3 | INRA-63 | 58087203 | 4.022 | 1.10 | 0.17 |
| 3 | INRA-353 | 58113454 | 4.383 | 1.00 | 0.17 |
| 3 | ARS-BFGL-NGS-32229 | 58265999 | 4.288 | 1.22 | 0.18 |
| 3 | Hapmap43441-BTA-103289 | 58630881 | 3.877 | 0.10 | 0.26 |
| 3 | INRA-451 | 59788079 | 3.951 | 0.65 | 0.2 |
| 3 | Hapmap41468-BTA-19097 | 60691443 | 2.915 | 0.25 | 0.26 |
| 3 | BTA-22670-no-rs | 61057371 | 5.052 | 0.09 | 0.26 |
| 3 | BFGL-NGS-111240 | 61234556 | 2.771 | 0.71 | 0.28 |
| 3 | BTB-00130588 | 61265255 | 4.351 | 0.78 | 0.32 |
| 3 | INRA-456 | 62008461 | 2.739 | 1.18 | 0.48 |
| 3 | BTA-10440-no-rs | 70663590 | 3.382 | 0.13 | 0.12 |
| 4 | BTA-87133-no-rs | 82332132 | 3.033 | 0.51 | 0.08 |
| 5 | UA-IFASA-5221 | 19015098 | 2.827 | - | 0.33 |
| 5 | Hapmap43960-BTA-85875 | 21211391 | 2.833 | - | 0.38 |
| 5 | ARS-BFGL-NGS-16315 | 21309336 | 3.292 | - | 0.18 |
| 5 | BTA-72805-no-rs | 21339472 | 2.74 | - | 0.31 |
| 5 | Hapmap44614-BTA-72802 | 21397136 | 2.953 | - | 0.40 |
| 5 | ARS-BFGL-NGS-54735 | 21603090 | 4.765 | - | 0.25 |
| 6 | ARS-BFGL-NGS-58275 | 48667759 | 2.927 | - | 0.08 |
| 6 | Hapmap47902-BTA-26537 | 50793466 | 2.711 | - | 0.08 |
| 6 | Hapmap40151-BTA-94687 | 55084058 | 3.51 | 0.58 | 0.10 |
| 6 | BTB-01133266 | 55213615 | 3.296 | 0.46 | 0.10 |
| 6 | BTA-94706-no-rs | 55247167 | 3.291 | 0.41 | 0.10 |
| 6 | Hapmap55334-rs29009712 | 56004491 | 3.025 | - | 0.09 |
| 6 | ARS-BFGL-NGS-103013 | 62309005 | 2.736 | 0.70 | 0.26 |
| 6 | BTB-01362502 | 65587693 | 2.812 | 1.83 | 0.34 |
| 6 | BTB-01941442 | 65968144 | 2.738 | 1.71 | 0.34 |
| 6 | BTB-01978479 | 66214474 | 2.78 | 1.77 | 0.34 |
| 7 | ARS-BFGL-NGS-14780 | 64185681 | 2.752 | 0.20 | 0.38 |
| 7 | BFGL-NGS-113819 | 64254252 | 3.121 | 0.97 | 0.32 |
| 7 | BFGL-NGS-109819 | 64308979 | 3.098 | 0.87 | 0.32 |
| 7 | BTA-88775-no-rs | 66885890 | 3.248 | 0.05 | 0.34 |
| 7 | ARS-BFGL-NGS-36796 | 83081854 | 2.886 | 1.34 | 0.26 |
| 7 | BTB-01321253 | 83116248 | 2.749 | 1.03 | 0.23 |
| 8 | BFGL-NGS-111488 | 65118526 | 3.499 | - | 0.08 |
| 8 | ARS-BFGL-NGS-24979 | 65518480 | 3.263 | - | 0.08 |
| 8 | ARS-BFGL-NGS-87805 | 66620157 | 2.958 | - | 0.07 |
| 8 | BTB-01530957 | 67496418 | 4.397 | - | 0.07 |
| 8 | BTB-01593847 | 78695447 | 4.491 | 0.96 | 0.18 |
| 8 | BTB-01593897 | 78722233 | 3.598 | - | 0.12 |
| 8 | ARS-BFGL-NGS-11101 | 80060251 | 3.202 | - | 0.13 |
| 8 | ARS-BFGL-NGS-15117 | 80417427 | 2.925 | - | 0.13 |
| 9 | ARS-BFGL-NGS-45802 | 86209499 | 3.023 | 0.71 | 0.07 |
| 9 | Hapmap41585-BTA-56206 | 88123447 | 2.785 | 0.92 | 0.05 |
| 9 | ARS-BFGL-NGS-101711 | 91588318 | 3.721 | 0.10 | 0.05 |
| 9 | ARS-BFGL-NGS-57866 | 92516448 | 3.218 | 0.47 | 0.33 |
| 9 | BTA-84833-no-rs | 93640208 | 3.325 | 0.02 | 0.29 |
| 9 | ARS-BFGL-NGS-58059 | 94492636 | 3.414 | 0.61 | 0.32 |
| 9 | Hapmap48098-BTA-84937 | 95541188 | 2.897 | 0.76 | 0.39 |
| 9 | Hapmap58613-rs29012081 | 95595087 | 2.897 | 0.82 | 0.39 |
| 9 | ARS-BFGL-NGS-40929 | 95692013 | 2.712 | 0.71 | 0.35 |
| 9 | Hapmap54387-rs29020464 | 96739032 | 2.751 | 1.14 | 0.42 |
| 10 | BTA-111053-no-rs | 98234834 | 2.939 | 0.25 | 0.42 |
| 12 | ARS-BFGL-BAC-14990 | 68809425 | 2.707 | 0.71 | 0.12 |
| 12 | BTB-00503574 | 69740098 | 3.127 | 0.28 | 0.19 |
| 12 | ARS-BFGL-NGS-59515 | 69872202 | 3.036 | 0.65 | 0.22 |
| 12 | BTA-29998-no-rs | 75672587 | 3.765 | 0.25 | 0.16 |
| 12 | ARS-BFGL-NGS-15699 | 75696505 | 3.342 | 0.44 | 0.2 |
| 12 | ARS-BFGL-NGS-16052 | 75901967 | 2.744 | 1.01 | 0.22 |
| 13 | BTB-01390401 | 83842783 | 3.357 | - | 0.26 |
| 13 | Hapmap40030-BTA-87432 | 83887570 | 2.976 | - | 0.26 |
| 14 | Hapmap46735-BTA-86653 | 26115329 | 2.728 | 0.05 | 0.36 |
| 15 | BTA-29693-no-rs | 48655849 | 2.834 | 1.08 | 0.43 |
| 15 | BTB-01372747 | 48965012 | 3.426 | - | 0.34 |
| 15 | BTA-36962-no-rs | 49187040 | 2.738 | 0.51 | 0.29 |
| 15 | Hapmap44373-BTA-36964 | 49214719 | 3.363 | 0.51 | 0.28 |
| 15 | Hapmap47590-BTA-36983 | 49448876 | 3.37 | 0.33 | 0.42 |
| 15 | BTB-02015886 | 50934058 | 2.927 | 0.74 | 0.45 |
| 15 | ARS-BFGL-BAC-19992 | 57827724 | 2.726 | 0.94 | 0.23 |
| 15 | ARS-BFGL-NGS-55206 | 58839306 | 3.648 | 1.51 | 0.28 |
| 15 | BTB-00607669 | 59213466 | 2.79 | 0.84 | 0.44 |
| 15 | ARS-BFGL-NGS-37842 | 59861111 | 4.004 | 0.72 | 0.31 |
| 15 | Hapmap42006-BTA-91398 | 61111954 | 3.196 | 0.29 | 0.38 |
| 15 | BTB-01059544 | 61305543 | 2.742 | 0.33 | 0.37 |
| 15 | Hapmap38148-BTA-27068 | 62887802 | 4.17 | **2.23** | 0.20 |
| 15 | BFGL-NGS-111146 | 63517478 | 3.083 | 0.83 | 0.45 |
| 15 | ARS-BFGL-NGS-63905 | 63624286 | 3.953 | 1.61 | 0.22 |
| 15 | BFGL-NGS-117803 | 63658321 | 3.977 | 1.64 | 0.23 |
| 15 | BTA-01263-rs29012228 | 64516076 | 3.597 | - | 0.2 |
| 15 | Hapmap48636-BTA-118794 | 64609636 | 2.901 | 0.06 | 0.43 |
| 15 | Hapmap57542-rs29012408 | 64644803 | 3.242 | 1.62 | 0.25 |
| 15 | BTB-01706506 | 64672104 | 3.009 | 0.06 | 0.43 |
| 15 | Hapmap59484-rs29019823 | 64779261 | 3.115 | 1.44 | 0.25 |
| 15 | BTA-37249-no-rs | 64988473 | 2.905 | 2.13 | 0.21 |
| 15 | BTB-00609706 | 65289691 | 3.995 | 1.18 | 0.24 |
| 15 | Hapmap41848-BTA-37274 | 65330022 | 4.271 | 0.88 | 0.48 |
| 15 | BFGL-NGS-115610 | 65436376 | 3.947 | 0.87 | 0.48 |
| 15 | Hapmap56240-ss46526743 | 65580889 | 3.821 | 0.81 | 0.49 |
| 15 | ARS-BFGL-NGS-105277 | 65614519 | 3.821 | 0.81 | 0.47 |
| 15 | ARS-BFGL-NGS-109573 | 65780389 | 3.522 | 0.81 | 0.47 |
| 15 | ARS-BFGL-NGS-82082 | 65812401 | 2.981 | 1.03 | 0.26 |
| 15 | BFGL-NGS-111210 | 65938662 | 2.742 | 0.17 | 0.39 |
| 15 | ARS-BFGL-NGS-15460 | 65962529 | 3.377 | 0.55 | 0.29 |
| 15 | Hapmap50340-BTA-37383 | 66243232 | 2.811 | 0.18 | 0.38 |
| 15 | BFGL-NGS-112642 | 66585363 | 3.302 | 0.48 | 0.29 |
| 15 | ARS-BFGL-NGS-32103 | 66623248 | 2.895 | 1.00 | 0.26 |
| 15 | BFGL-NGS-116120 | 68181603 | 2.835 | 1.01 | 0.44 |
| 15 | ARS-BFGL-NGS-106323 | 68198405 | 2.792 | 1.02 | 0.44 |
| 16 | ARS-BFGL-NGS-1082 | 50147154 | 2.95 | 0.48 | 0.12 |
| 22 | ARS-BFGL-NGS-68194 | 58575100 | 2.84 | - | 0.14 |
| 25 | ARS-BFGL-NGS-7215 | 20799329 | 3.068 | 0.14 | 0.46 |
| 26 | BTB-01716946 | 12849949 | 2.736 | 1.08 | 0.21 |
| 27 | BTB-02001456 | 9696969 | 3.529 | 0.04 | 0.21 |
| 27 | ARS-BFGL-NGS-13449 | 37755815 | 2.736 | - | 0.10 |
| 27 | ARS-BFGL-NGS-73826 | 38439988 | 3.273 | - | 0.13 |
| 27 | ARS-BFGL-NGS-45270 | 38674327 | 4.61 | 0.81 | 0.31 |
| 27 | ARS-BFGL-NGS-58720 | 39016769 | 4.887 | 0.59 | 0.27 |
| 27 | Hapmap58237-rs29017248 | 39063966 | 4.518 | 0.50 | 0.27 |

^*^iHS is not shown (-) when the value is not available.
