# Supplementary material for: Genome-Wide Scan of Gastrointestinal Nematode Resistance in Closed Angus Population Selected for Minimized Influence of MHC
Source: PLoS One. 2015 Mar 24;10(3):e0119380. doi: 10.1371/journal.pone.0119380 (PMC4372334; doi:10.1371/journal.pone.0119380)
Supplement: S2 Table — (DOCX) [file pone.0119380.s008.docx]

**S2 Table. Genes related to immune response in genomic regions under selection (|iHS|>3).**

| **ID^*^** | **BTA** | **Region (Mb)** | **Gene name / Function** |
| --- | --- | --- | --- |
| *GNG5* | 3 | 63.12-63.13 | similar to guanine nucleotide binding protein (G protein), gamma 5; guanine nucleotide binding protein (G protein), gamma 5  **Chemokine signaling pathway** |
| *PRKACB* | 3 | 63.41-63.57 | protein kinase, cAMP-dependent, catalytic, alpha; protein kinase,  cAMP-dependent, catalytic, beta  **Chemokine signaling pathway** |
| *GRK4* | 6 | 107.84-107.89 | G protein-coupled receptor kinase 4  **Chemokine signaling pathway** |
| *PIK3R2* | 7 | 49.87-49.99 | phosphoinositide-3-kinase, regulatory subunit 2 (beta)  **Chemokine signaling pathway** |
| *IL12RB1* | 7 | 50.51-50.68 | interleukin 12 receptor, beta 1  **Cytokine-cytokine receptor interaction** |
| *SYK* | 8 | 88.42-88.52 | spleen tyrosine kinase  **Natural killer cell mediated cytotoxicity,**  **B cell receptor signaling pathway** |
| *RASGRP1* | 10 | 33.73-33.81 | RAS guanyl releasing protein 1 (calcium and DAG-regulated)  **T cell receptor signaling pathway** |
| *PAK6* | 10 | 35.67-35.70 | p21 protein (Cdc42/Rac)-activated kinase 6  **T cell receptor signaling pathway** |
| *ARF6* | 10 | 42.64-42.64 | ADP-ribosylation factor 6  **Fc gamma R-mediated phagocytosis** |
| *SOS2* | 10 | 42.83-42.95 | similar to son of sevenless homolog 2  **Chemokine signaling pathway** |
| *MARCKS-*  *like 1* | 12 | 33.31-33.32 | Macrophage myristoylated alanine-rich C kinase substrate like 1  **Fc gamma R-mediated phagocytosis** |
| *TNFRSF19* | 12 | 34.58-34.66 | tumor necrosis factor receptor superfamily, member 19  **Cytokine-cytokine receptor interaction** |

^*^ Genes on BTA23 are not included.
